# High frequency of complex *TP53* mutations in CNS metastases from breast cancer

**DOI:** 10.1038/bjc.2011.464

**Published:** 2011-12-20

**Authors:** C Lo Nigro, D Vivenza, M Monteverde, L Lattanzio, O Gojis, O Garrone, A Comino, M Merlano, P R Quinlan, N Syed, C A Purdie, A Thompson, C Palmieri, T Crook

**Affiliations:** 1 Laboratory of Cancer Genetics and Translational Oncology, Oncology Department, S Croce General Hospital, Cuneo, Italy; 2 Cancer Research UK Laboratories, Division of Cancer, Imperial College London-Hammersmith Campus, Du Cane Road, London W12 0NN, UK; 3 Medical Oncology, Oncology Department, S Croce General Hospital, Cuneo, Italy; 4 Department of Pathology, S Croce General Hospital, Cuneo, Italy; 5 Dundee Cancer Centre, University of Dundee, Ninewells Hospital, Dundee DD1 9SY, UK; 6 John Fulcher Neuro-Oncology Laboratory, Faculty of Medicine, Imperial College London, Neuroscience Centre, Charing Cross Hospital, London W6 8RF, UK

**Keywords:** breast cancer, CNS metastasis, p53, triple-negative breast cancer

## Abstract

**Background:**

Brain metastasis from breast cancer is usually associated with a poor prognosis and early death. Alteration of p53 may contribute to malignant progression by abrogation of apoptosis induced by oncogene activation and by acquisition of gain-of-function properties, which promote tumour aggression. Mutation in *TP53* occurs at high frequency in carcinomas of the lung and gastro-intestinal tract, but is much less frequent, at 25%, in primary breast cancer. The frequency of *TP53* alteration in the central nervous system (CNS) metastatic breast cancer is not known.

**Methods:**

In all, 23 cases of histologically confirmed CNS metastatic breast cancer were identified and the coding sequence of *TP53* determined. *TP53* was also sequenced in two control series of primary breast carcinomas from independent clinical centres.

**Results:**

We demonstrate a strikingly high frequency of *TP53* mutation in the CNS metastatic lesions with an over-representation of complex mutations (non-sense/deletions/insertions). Complex mutations occur in metastatic lesions in both triple-negative breast cancer and hormone receptor/HER2-positive cases. Analysis of paired primary carcinomas and brain metastatic lesions revealed evidence for both clonal selection and generation of new mutations (missense and complex) in progression from a primary breast carcinoma to brain metastasis.

**Conclusion:**

Mutation in *TP53* is the most common genetic alteration reported during metastasis to the brain in breast cancer.

To all intents and purposes, development of metastatic disease implies that breast cancer is no longer curable. A survey of the literature shows that almost any distant organ or tissue can be the site of metastatic disease, but certain organs namely bone, lung, liver and brain are particularly common sites of such disease dissemination. Central nervous system (CNS) metastasis is the most common type of malignancy found in the brain and breast cancer is the second most common type of malignancy to cause CNS metastases (Sharma and Abraham, 2007). Central nervous system metastases are less common than bone or visceral metastases, but are less sensitive to systemic chemotherapy and are associated with significantly worse clinical outcomes. Recently, a trend towards increasing CNS recurrence has been noted, up to 25–34% as compared with historical rates of 1–16%. This may be due to increased use of sensitive detection methods such as contrast-enhanced magnetic resonance imaging, increased awareness by patients and clinicians, or an alteration of the natural history of breast cancer resulting from improvements in systemic therapies that are prolonging survival. Recent studies have revealed that breast cancer subtypes are associated with distinct patterns of metastatic spread, with luminal/HER2, HER2-enriched and basal-like tumours having a higher rate of brain metastasis. These subtypes are associated with a poorer prognosis (Kennecke *et al*, 2010). Two studies have sought to define changes in gene expression, which associate with brain metastasis in breast cancer and a number of candidate genes whose expression is up- or downregulated in the CNS metastatic lesions relative to the primary lesion have been identified (Bos *et al*, 2009; Klein *et al*, 2009).

Alteration of p53 by various mechanisms is a frequent finding in malignant disease, although the frequency of mutation in the gene varies considerably between tumour types, with a high proportion of mutations in lung cancer, lower gastrointestinal cancers and glial brain tumours, but low reported frequencies in melanoma and sarcomas (Soussi and Wiman, 2007). In breast cancer, an overall frequency of approximately 20% has been documented (Pharoah *et al*, 1999; Olivier *et al*, 2006). Many *TP53* mutations identified in human cancer encode mutant proteins, which possess gain of function, such as the ability to cooperate with activated oncogenes to morphologically transform primary cells. The most common missense mutations in human cancer are termed ‘hotspot mutations’ (Walker *et al*, 1999). More complex mutations such as insertion/deletion/nonsense are also described. In breast cancer, the frequency of *TP53* mutation is higher in carcinomas arising in a background of mutant BRCA1 and BRCA2 than in sporadic cases (Crook *et al*, 1997; Smith *et al*, 1999). Complex mutations appear more common in cancers arising in cancers of basal subtype, including but not restricted to those arising in a background of mutant BRCA1 (Holstege *et al*, 2009; Manié *et al*, 2009). Studies of *TP53* mutations in metastatic breast cancer lesions have been limited by the lack of availability of tissue, perhaps because of the infrequency of re-biopsy of radiologically detected suspicious lesions in patients with a previous history of primary breast cancer. However, Ding *et al* (2010) used massively parallel sequencing to examine genetic changes in a primary breast carcinoma and subsequent CNS metastasis and identified a complex mutation in *TP53*. Here, we have analysed *TP53* mutations in a series of CNS metastatic breast carcinomas.

## Materials and methods

### Tumours

Case ascertainment of surgically excised and histologically confirmed intra-cranial brain metastatic breast cancer identified 23 metastases subsequently retrieved from the neuropathology archives of the Charing Cross Hospital. As comparator primary breast cancers, we analysed using different sequencing techniques *TP53* mutation in independent series of primary breast carcinomas from two different clinical centres. In each series, expression of the oestrogen receptor (ER), progesterone receptor (PR) and HER2 was performed according to standard protocols of clinical care (Purdie *et al*, 2010).

### *TP53* sequence analysis

Genomic DNA was isolated from archival cases by proteinase K digestion of 10 *μ*M formalin-fixed, paraffin-embedded (FFPE) sections using a standard xylene–phenol protocol. For the primary breast cancers in the Tayside Tissue Bank (Dundee, UK), genomic DNA was isolated from frozen tissues as described previously (Baker *et al*, 2010). Mutations in *TP53* were detected using two methods. For the Tasyide cases, *TP53* was analysed using the Roche Amplichip (Roche Molecular Systems, Pleasanton, CA, USA) as reported previously (Baker *et al*, 2010). For the CNS metastatic lesions and the FFPE cases, *TP53* was analysed by direct sequencing of exons 4–10, which were individually amplified (Table 1). Polymerase chain reaction was carried out in a total volume of 10 *μ*l consisting of 0.75 *μ*M of both primers, 1.5 mmol l^−1^ MgCl_2_, 200 *μ*M deoxynucleotide triphosphate, 0.25 U of AmpliTaq Gold 360 DNA Polymerase (Applied Biosystems, Foster City, CA, USA), 1 *μ*l of 360 GC Enhancer, 50 ng of genomic DNA using a GeneAmp PCR System 9700 Thermal Cycler (Applied Biosystems). The PCR programme was an initial denaturation step of 10 min at 95 °C, followed by 40 cycles of 30 s at 95 °C, 45 s at specific annealing temperature for each fragment and 45 s at 72 °C, with a final step at 72 °c for 7 min. Amplified products of p53 exons (4–10) were directly sequenced in both directions on an ABI 3130 automated sequencer, using the BigDye terminator cycle sequencing reaction kit following the manufacturer's instructions (Applied Biosystem). The analysis was performed using the Variant Reporter Software v.1.0 (Applied Biosystems). Proposed mutations were confirmed on both strands on repeated, independent amplimers and validated by the IARC *TP53* Mutation Database (http://www-p53.iarc.fr). The reference sequence is the genomic sequence NC_000017 version 9 (7512445–7531642) from GenBank.

## Results

### *TP53* mutations in CNS metastatic breast cancer

**Figure 1:**
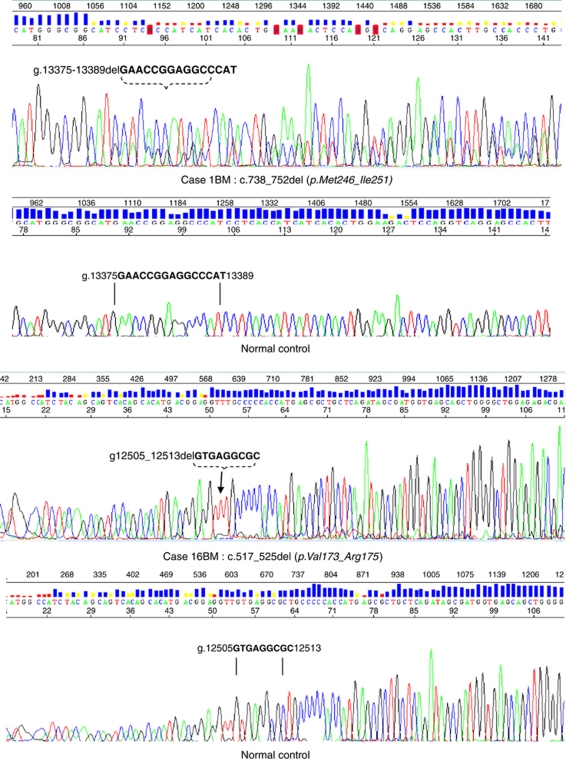
Representative sequencing traces showing two examples of complex *TP53* mutations in central nervous system (CNS) metastatic breast cancer lesions. Isolation of genomic DNA, sequencing and analysis were carried out as described in materials and methods. The top panel shows Case 1BM with a normal control sequencing trace immediately below. The second case is 16BM, again with a normal control immediately below. The deleted sequence is indicated by vertical lines.

We analysed the genomic sequence of *TP53* in 23 individual cases of surgically resected, histologically confirmed CNS metastatic breast cancer. As controls, we sequenced two independent series of primary breast carcinomas from two different oncology centres using two different techniques. *TP53* mutations were present in 29 out of 91 (32%) cases from northern Italy (series 1) and in 55 out of 229 (24%) cases from the Tayside series (series 2). In the CNS metastatic lesions, mutations were present in 20 out of 23 (87%) cases (Figure 1 and Tables 2a and b). Two different *TP53* mutations were detected in 2 out of 23 CNS metastatic lesions, 4 out of 29 primary carcinomas from series 1 and 0 out of 55 primary carcinomas from series 2 (Tables 2). In comparator series 1, 25 out of 87 ER-positive and 4 out of 4 ER-negative cases contained *TP53* mutations. In series 2, *TP53* mutations were present in 27 out of 177 ER-positive and 27 out of 52 ER-negative cases. The ER status was unavailable for one Her2-positive case (55P) with a *TP53* mutation and for two ER-negative cases with *TP53* mutations (37P and 38P). Mutation in *TP53* was detected in 16 out of 34 triple-negative breast cancer (TNBC) in series 2. Frameshift, splice and nonsense mutations and in-frame insertions and deletions constitute complex *TP53* mutations (Holstege *et al*, 2009). Out of 22 (41%) mutations, nine detected in the CNS metastases were complex and such mutations were present in 9 out of 20 (45%) such cases, compared with 0 out of 34 (0%) complex mutations in primary carcinomas from comparator series 1 and 7 out of 55 (13%) complex mutations in primary carcinomas from comparator series 2. In the CNS metastatic lesions, 13 mutations caused amino-acid substitutions and 9 out of 13 (69%) were hotspot mutations (Tables 2a and b). Hotspot mutations comprised 11 out of 34 (32%) substitution mutations in comparator series 1 (Tables 2c and d) and 30 out of 48 substitution mutations (62%) in comparator series 2 (Tables 2e and f). The frequency of hotspot mutations was not significantly different between primary cancers, which subsequently relapsed with metastasis and those which did not, in either series 1 or series 2. In the CNS metastases, complex mutations were present in both TNBC and hormone receptor cases: 6 out of 9 complex mutations occurred in hormone receptor-positive cancers compared to 3 out of 9 in TNBC. In comparator series 2, 6 out of 7 complex mutations were in ER-negative cases, of which four were TNBC.

### Analysis of paired primary/metastatic lesions reveals clonal selection for loss of p53 function and generation of new mutations

**Figure 2:**
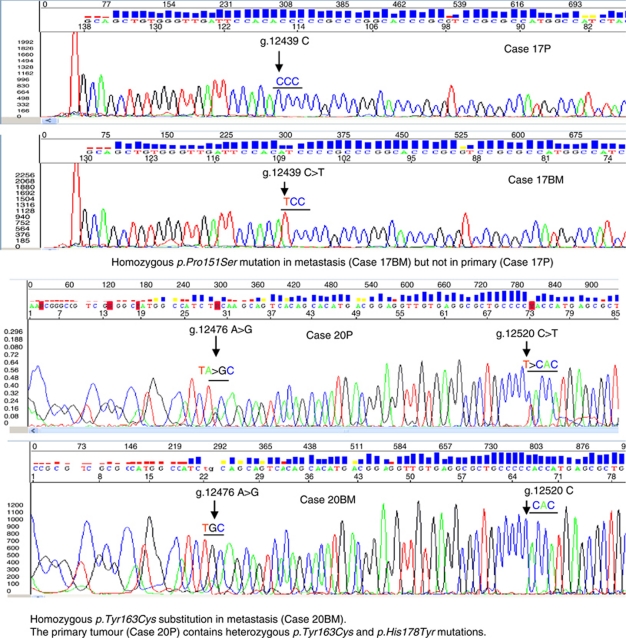
Representative sequencing traces showing *TP53* mutations in matched primary and central nervous system (CNS) metastatic breast cancer lesions. Isolation of genomic DNA, sequencing and analysis were carried out as described in Materials and Methods. The upper panels show homozygous mutation *Pro151Ser* in CNS metastasis 17BM (arrowed), but only wild-type sequence in the primary carcinoma (17P). The lower panels show heterozygous *Tyr163Cys* and *p.His178Tyr* mutations (arrowed) in the primary carcinoma, 20P, but homozygous *Tyr163Cys* mutation (arrowed) in the CNS metastasis (20BM).

The *TP53* mutation frequency in comparator series 1 is 9 out of 30 (30%) in non-relapsed cases and 20 out of 61 (33%) in relapsed cases, and in comparator series 2, it is 29 out of 161 (18%) in non-relapsed cases and 26 out of 68 (38%) in relapsed cases. This implies that *TP53* mutation frequency is higher in CNS metastatic lesions than in primary breast carcinomas, irrespective of whether the primary lesion ultimately relapsed. To further investigate this, we analysed four paired cases with tissue from both primary and CNS metastasis and we sequenced *TP53* to determine whether mutations associated with subsequent CNS metastasis were detectable in primary cancers (Figure 2). In all four cases, *TP53* mutations were present in the metastatic lesion (Table 2). In two of the four cases, the *TP53* mutation present as the sole variant in the CNS metastasis was also detected in the primary cancer, in both cases in the presence of other mutations: in Case 19P, three mutations (*Pro300Leu*, *Ser260Phe* and the complex mutation *Ser183Ter*) were detected, but only *Ser183Ter* (homozygous) was detected in the CNS metastasis 19BM. In Case 20P, *His178Tyr* and *Tyr163Cys* were present, but only the hotspot mutation *Tyr163Cys* was detected (homozygous) in the CNS metastasis 20BM. In a third case (17P), no mutations were detected in the primary cancer, but hotspot mutation *Pro151Ser* was present as a homozygous mutation in the CNS metastasis (17BM). In Case 18, *Ile232Phe* and *Tyr234His* were detected in the primary (18P), but a new mutation *Val216Met* was detected in the CNS metastasis (18BM).

## Discussion

Dissemination of breast cancer to the brain is a sinister event, associated with a poor prognosis. Treatment of such metastatic lesions is typically with radiotherapy, although solitary lesions can be resected, and in some cases an improved prognosis is observed. Nonetheless, the poor outlook and resistance to chemotherapy seen in most reported series strongly emphasises the need for improved understanding of the mechanisms of brain metastasis and for novel therapeutic approaches. Studies of the molecular basis of metastasis of breast carcinomas to the brain have been limited. Changes in gene expression in CNS metastatic lesions in breast cancer patients have been reported in two clinical studies (Nishizuka *et al*, 2002; Klein *et al*, 2009) and a pre-clinical study analysed changes in gene expression in MDA MB 231 cells with tropism for metastasis to the brain (Bos *et al*, 2009). Here, we have analysed the coding sequence of *TP53* in a series of brain metastases, confirmed by histopathology to derive from primary breast carcinomas, and we report a high frequency of *TP53* mutational alteration in breast cancer metastatic to the brain. This demonstrates that mutation of *TP53* is the most common change (genetic or epigenetic) identified thus far in brain metastases from breast cancer.

As comparators for the metastatic brain lesions, we tested two independent series of primary breast carcinomas from geographically distinct clinical practices. One series comprised archival cases in which genomic DNA was prepared from FFPE tissue and the other series high-molecular-weight genomic DNA from fresh–frozen tissue. Cases were evaluated for tumour cell representation before analysis. The overall frequency of *TP53* mutation detected in each series was 32 and 23%, with a low frequency of complex mutations, comparable to those reported in previous studies (Pharoah *et al*, 1999; Olivier *et al*, 2006). There have been very few previous studies of *TP53* in CNS metastatic breast cancer lesions. However, our data are consistent with Ding *et al* (2010), who reported a frameshift mutation present in both primary and CNS metastasis of a basal phenotype breast cancer. Our results are also consistent with those of Piccirilli *et al* (1994), who showed 17p loss of heterozygosity and *TP53* mutations in breast carcinomas metastatic to brain. Further, immunoreactivity for p53 in primary breast carcinomas, using antibodies pAb240 and pAb1801, is associated with increased risk for subsequent brain metastasis (Tham *et al*, 2006), consistent with a potential role for *TP53* mutation in CNS metastasis. The frequency of complex mutations in the CNS metastatic lesions is higher than that in either of the series of primary carcinomas. The frequency of complex mutations is reported to be higher in basal subtype breast cancers, including cases arising in carriers of germ-line BRCA1 mutations (Holstege *et al*, 2009; Manié *et al*, 2009). In our series of CNS metastases, 7 out of 10 TNBC contained missense rather than complex mutations. Furthermore, 6 out of 9 complex mutations occurred in hormone receptor-positive cancers compared to 3 out of 9 in TNBC, implying that the high frequency of complex mutations in the CNS metastatic lesions cannot be explained on the basis of over-representation of TNBC. Analysis of matched primary and CNS metastatic lesions revealed selection of mutants during metastasis to the brain. For example, in one case, three *TP53* mutations were detected in the primary lesion, but only the complex mutation resulting in premature termination at codon 183 was detected in the CNS metastasis from this patient (as a homozygous mutation), showing preferential selection of the complex mutation. A second primary cancer contained two detectable *TP53* mutations, a non-hotspot *His178Tyr* and the hotspot *Tyr163Cys*. Only the hotspot mutation was detected (again homozygous) in the CNS metastasis.

In previous studies, conservation of *TP53* mutation between primary and paired CNS metastasis was reported (Piccirilli *et al*, 1994). In another study, the same single mutation was detected in the primary breast carcinoma and paired metastatic lymph node in a subset of cases. In other cases, an additional mutation in the primary tumour only or a mutation in the metastasis only was observed (Peller *et al*, 1995). In our series, the final, clonal *TP53* mutation was only detected in the primary lesion in two of four cases, and in both such cases, additional mutations were present in the primary lesion. We did not observe association between specific missense *TP53* mutations and metastasis to CNS or other anatomical sites in the clinical series analysed. Rather, the common property in all such cases from series 2, which progressed to CNS metastatic disease was overexpression of Pin1 (data not shown), consistent with the critical role of this protein in promoting aggressive clinical phenotypes in breast cancers expressing mutant p53 (Girardini *et al*, 2011).

What are the implications of our data? First, the presence of *TP53* mutations in the great majority of CNS metastatic lesions implies that therapeutic strategies aimed at exploiting loss of p53 function could be highly effective in brain metastasis, even if the primary breast cancer is wild type for *TP53*. Second, the results raise the possibility that patients at high risk for intra-cranial metastasis might be identifiable from detection of *TP53* mutations in the primary cancer and interventions such as prophylactic cranial irradiation considered. However, our analysis of paired primary, metastatic cases, albeit with limited numbers, shows that *TP53* mutations present in brain metastatic lesions are not always detectable in the primary. Also, despite the increased representation of complex mutations, many CNS metastases nonetheless occurred in cancers containing hotspot (substitution) mutations in *TP53*, reducing the likely sensitivity of detection of complex mutations as predictors of future CNS metastasis. As noted above, all primary cancers in series 2, which contained substitution mutations in *TP53*, and which subsequently progressed to CNS metastasis, overexpressed Pin1. Additional studies of the prognostic utility of Pin1 and mutant p53 are merited clearly. Third, our results further add to the evidence that changes in expression and structure of genes may occur in the progression of primary breast cancer to metastatic disease. In conclusion, our data reveal a strikingly high frequency of *TP53* mutation in CNS metastatic breast cancer.

## Figures and Tables

**Table 1:** Primers and conditions for analysis of p53

***TP53***	**Primer sequences**	**Product size (bp)**	***T* annealing (°C)**
4MutP53Fw1	5′-AGGACCTGGTCCTCTGACTGC-3′	154	61
4MutP53Rev1	5′-AGCAGCCTCTGGCATTCTGG-3′		
4MutP53Fw2	5′-AGAATGCCAGAGGCTGCTCC-3′	192	58
4MutP53Rev2	5′-GCAACTGACCGTGCAAGTCA-3′		
5MutP53Fw1	5′-TTATCTGTTCACTTGTGCC-3′	130	52
5MutP53Rev1	5′-TGTGGAATCAACCCACAGC-3′		
5MutP53Fw2	5′-GCAGCTGTGGGTTGATTCC-3′	166	58
5MutP53Rev2	5′-CCAGCCCTGTCGTCTCTCCA-3′		
6MutP53Fw	5′-GGCCTCTGATTCCTCACTGA-3′	199	58
6MutP53Rev	5′-GCCACTGACAACCACCCTTA-3′		
7MutP53Fw	5′-TGCCACAGGTCTCCCCAAGG-3′	196	56
7MutP53Rev	5′-AGTGTGCAGGGTGGCAAGTG-3′		
8MutP53Fw	5′-CCTTACTGCCTCTTGCTTCT-3′	225	58
8MutP53Rev	5′-ATAACTGCACCCTTGGTCTC-3′		
9MutP53Fw	5′-GCCTCAGATTCACTTTTATCACC-3′	152	56
9MutP53Rev	5′-CTTTCCACTTGATAAGAGGTCCC-3′		
10MutP53Fw	5′-CAGGTACTGTGTATATACTTACTTCTCC-3′	199	55
10MutP53Rev	5′-AGGAAGGCAGGGGAGTAGG-3′		

**Table 2:** *TP53* mutations in primary (P) and brain metastatic (BM) breast carcinomas

**(A) CNS metastatic lesions without matched primary (16 cases, 18 mutations)**				
**Case**	**Receptor status**	***TP53* sequence** a	**Type of mutation**	**Protein consequence**
1BM	TNBC	**c.738_752del**	**Deletion**	**p.Met246_Ile251**
2BM	TNBC	c.742C>T	Substitution	*p.Arg248Trp* (D)
3BM	TNBC	c.392A>T	Substitution	*p.Asn131Ile* (D)
4BM	TNBC	**c.393_395del**	**Deletion**	**p.Asn131_Lys132** b
5BM	TNBC	c.830G>T	Substitution	*p.Cys277Phe* (D)
6BM	TNBC	c.743G>A	Substitution	*p.Arg248Gln* (D)
7BM	TNBC	c.818G>A^c^	Substitution	*p.Arg273His* (D)
8BM	ER− PR− Her2+	c.743G>A^c^	Substitution	*p.Arg248Gln* (D)
9BM	ER− PR− Her2+	c.517G>A	Substitution	*p.Val173Met* (D)
10BM	ER− PR− Her2+	**c.820_821insT**	**Insertion**	p.Val274fs*305
11BM	ER+ PR− Her2−	c.743G>A	Substitution	*p.Arg248Gln* (D)
12BM	ER+ PR− Her2−	**c.812_820del**	**Deletion**	**p.Glu271_Val274**
13BM	ER+ PR− Her2−	**c.965delC** c	**Deletion**	p.Pro322Hisfs*344
14BM	ER+ PR+ Her2−	**c.450_451insC**	**Insertion**	p.Pro151fs*180
15BM	ER+ PR+ Her2−	**c.820_821insT** c	**Insertion**	p. Val274fs*305
		c.746G>A	Substitution	*p.Arg249Lys* (D)
16BM	ER+ PR+ Her2+	**c.517_525del**	**Deletion**	**p.Val173_Arg175**
		c.1082G>A	Substitution	*p.Gly361Glu* (N)

**(B) CNS metastatic lesions with matched primary (4 cases, 7 mutations in primary; 4 mutations in metastases)**				
**Case**	**Receptor status**	***TP53* sequence** a	**Type of mutation**	**Protein change**
17P	TNBC	Wild type		
17BM	TNBC	c.463A>T^c^	Substitution	*p.Pro151Ser* (D)
18P	TNBC	c.694A>T	Substitution	*p.Ile232Phe* (D)
		c.700T>C	Substitution	*p.Tyr234His* (D)
18BM	TNBC	c.646G>A	Substitution	*p.Val216Met* (D)
19P	TNBC	c.548C>G^c^	**Substitution**	**p.Ser183Ter**
		c.779C>T	Substitution	*p. Ser260Phe* (N)
		c.899C>T	Substitution	*p. Pro300Leu* (N/D)
19BM	TNBC	c.548C>G	**Substitution**	**p.Ser183Ter**
20P	ER− PR+ Her2−	c.488A>G	Substitution	*p.Tyr163Cys* (D)
		c.532C>T	Substitution	*p.His178Tyr* (D)
20BM	ER− PR+ Her2−	c.488A>G^c^	Substitution	*p.Tyr163Cys* (D)

**(C) Series 1 (S1). Primary lesions with metastatic relapse: 20 cases, 24 mutations**					
**Case**	**Receptor status**	***TP53* sequence** a	**Type of mutation**	**Protein change**	**Metastatic site**
S1 1P	TNBC	c.422G>T	Substitution	*p.Cys141Phe* (D)	LN, Pl
S1 2P	ER− PR− Her2+	c.422G>T	Substitution	*p.Cys141Phe* (D)	Lu, Li
S1 3P	ER− PR− Her2+	c.524G>A	Substitution	*p.Arg175His* (D)	Li, Pl
S1 4P	ER− PR− Her2+	c.339C>A	Substitution	*p.Phe113Leu* (D)	Ax
		c.853G>A	Substitution	*p.Glu285Lys* (D)	
S1 5P	ER+ PR− Her2−	c.673G>A	Substitution	*p.Val225Ile* (N)	Bo, Lu, Pl
S1 6P	ER+ PR− Her2−	c.524G>A	Substitution	*p.Arg175His* (D)	Bo, Lu, Pl
S1 7P	ER+ PR− Her2−	c.353C>T	Substitution	*p.Thr118Ile* (D)	Lu, LN
S1 8P	ER+ PR− Her2−	c.889C>T	Substitution	*p.His297Tyr* (N)	Li, Pl
S1 9P	ER+ PR− Her2+	c.253C>T; c.254 C>T	Substitution	*p.Pro85Ser* (N)	Br, Li, Lu
		c.878G>A	Substitution	*p.Gly293Glu* (N)	
S1 10P	ER+ PR− Her2+	c.839G>A	Substitution	p.*Arg280Lys* (D)	Ch
S1 11P	ER+ PR+ Her2−	c.738G>A	Substitution	*p. Met246Ile* (D)	Bo, Li, Lu
		c.782G>A	Substitution	*p.Ser261Asn* (N)	
S1 12P	ER+ PR+ Her2−	c.305C>T	Substitution	*p. Thr102Ile* (N)	Bo, LN
S1 13P	ER+ PR+ Her2−	c.422G>T	Substitution	*p.Cys141Phe* (D)	Bo, Li
S1 14P	ER+ PR+ Her2−	c.232G>A	Substitution	*p.Ala78Thr* (N)	Li, Bo
S1 15P	ER+ PR+ Her2+	c.659A>G	Substitution	*p. Tyr220Cys* (D)	Li
S1 16P	ER+ PR+ Her2+	c.853G>A	Substitution	*p.Glu285Lys* (D)	Sc
S1 17P	ER+ PR+ NA	c.100C>T	Substitution	*p.Pro34Ser* (N)	Bo
		c.595G>A	Substitution	*p.Gly199Arg* (D)	
S1 18P	ER+ PR+ NA	c.964C>T	Substitution	*p.Pro322Ser* (N)	Bo
S1 19P	ER+ PR+ NA	c.380C>T	Substitution	*p.Ser127Phe* (D)	Bo, LN
S1 20P	ER+ PR+ NA	c.305C>T	Substitution	*p.Thr102Ile* (N)	St

**(D) Series 1 (S1). Primary lesions with no metastatic relapse: 9 cases, 11 mutations**				
**Case**	**Receptor status**	***TP53* sequence** a	**Type of mutation**	**Protein change**
S1 21P	ER+ PR+ Her2−	c.274C>T	Substitution	*p.Pro92Ser* (N)
		c.1075C>T	Substitution	*p.Pro359Leu* (D)
S1 22P	ER+ PR+ Her2−	c.677G>A	Substitution	*p.Gly226Asp* (N)
S1 23P	ER+ PR+ Her2−	c.872A>G;c.873G>A	Substitution	*p.Lys291Arg* (D)
S1 24P	ER+ PR+ Her2−	c.905G>A	Substitution	*p.Gly302Glu* (N)
S1 25P	ER+ PR+ Her2−	c.343C>T	Substitution	*p.His115Tyr* (D)
		c.1081G>A	Substitution	*p.Gly361Arg* (N)
S1 26P	ER+ PR− Her2−	c.524G>A	Substitution	*p.Arg175His* (D)
S1 27P	ER+ PR− Her2−	c.692C>T	Substitution	*p.Thr231Ile* (D)
S1 28P	ER+ PR− Her2−	c.823T>C	Substitution	*p.Cys275Arg* (D)
S1 29P	ER+ PR+ NA	c.832C>T	Substitution	*p.Pro278Ser* (D)

**(E) Series 2 (S2). Primary lesions (*N*=68) with metastatic relapse: 26 cases, 26 mutations**					
**Case**	**Receptor status**	***TP53* sequence**	**Type of mutation**	**Protein change**	**Metastatic site**
S2 1P	TNBC	c.796G>A	Substitution	*p.Gly266Arg* (D)	Br, Ch, Lu
S2 2P	TNBC	**c.981T>A**	**Substitution**	**p.Tyr327Ter**	Lu
S2 3P	TNBC	c.833C>A	Substitution	*p.Pro278His* (D)	As, Bo, Br, Lu
S2 4P	TNBC	c.536A>G	Substitution	*p.His179Arg* (D)	Bo, Lu
S2 5P	TNBC	c.517G>T	Substitution	*p.Val173Leu* (D)	Li, Lu
S2 6P	TNBC	**c.586C>T**	**Substitution**	**p.Arg196Ter**	Li, Lu
S2 7P	TNBC	c.452C>A	Substitution	*p.Pro151His* (D)	Lu
S2 8P	TNBC	c.706T>A	Substitution	*p.Tyr236Asn* (D)	Ch, skin
S2 9P	TNBC	c.820G>T	Substitution	*p.Val274Phe* (D)	Lu
S2 10P	ER− PR− Her2+	c.817C>T	Substitution	*p.Arg273Cys* (D)	Li
S2 11P	ER− PR− Her2+	c.524G>A	Substitution	*p.Arg175His* (D)	Bo, Br, Lu
S2 12P	ER− PR− Her2+	c.836G>A	Substitution	*p.Gly279Glu* (D)	Lymphangitis
S2 13P	ER− PR− Her2+	c.707A>G	Substitution	*p.Tyr236Cys* (D)	Br, Thy
S2 14P	ER− PR− Her2+	**c.178delC**	**Deletion**	p.Pro60Glnfr*122	Bo, Li, Lu
S2 15P	ER− PR− Her2+	c.395A>G	Substitution	*p. Lys132Arg* (D)	Ch, Li
S2 16P	ER+ PR− Her2−	c.1009C>G	Substitution	*p.Arg337Gly* (D)	Bo, Li
S2 17P	ER+ PR− Her2−	c.818G>A	Substitution	*p.Arg273His* (D)	Bo, Li
S2 18P	ER+ PR− Her2+	c.736A>G	Substitution	*p.Met246Val* (D)	LN
S2 19P	ER+ PR− Her2+	c.395A>G	Substitution	*p.Lys132Arg* (D)	Bo, Br, Ch, LN, Lu
S2 20P	ER+ PR+ Her2−	**c.574C>T**	**Substitution**	**p.Gln192Ter**	Bo, Lu
S2 21P	ER+ PR+ Her2−	c.646G>A	Substitution	*p.Val216Met* (D)	Bo, Br, Li
S2 22P	ER+ PR+ Her2−	c.389T>C	Substitution	*p.Leu130Pro* (D)	Bo
S2 23P	ER+ PR+ Her2−	c.536A>G	Substitution	*p.His179Arg* (D)	As
S2 24P	ER+ PR+ Her2+	c.738G>T	Substitution	*p.Met246Ile* (D)	Bo
S2 25P	ER+ PR+ Her2+	c.524G>A	Substitution	*p.Arg175His* (D)	Li
S2 26P	ER+ PR+ Her2+	c.785G>T	Substitution	*p.Gly262Val* (D)	Bo, Br, Ch

**(F) Series 2 (S2). Primary lesions with no metastatic relapse: 29 cases, 29 mutations**				
**Case**	**Receptor status**	***TP53* sequence**	**Mutation type**	**Protein change**
S2 27P	TNBC	**c.637C>T**	**Substitution**	**p.Arg213Ter**
S2 28P	TNBC	c.725G>A	Substitution	*p.Cys242Tyr* (D)
S2 29P	TNBC	c.724T>G	Substitution	*p.Cys242Gly* (D)
S2 30P	TNBC	c.817C>T	Substitution	*p.Arg273Cys* (D)
S2 31P	TNBC	c.396G>T	Substitution	*p.Lys132Asn* (D)
S2 32P	TNBC	c.536A>G	Substitution	*p.His179Arg* (D)
S2 33P	TNBC	**c.1024delC**	**Deletion**	p.Arg342Glufs*344
S2 34P	ER− PR− Her2+	**c.493C>T**	**Substitution**	**p.Gln165Ter**
S2 35P	ER− PR− Her2+	c.818G>A	Substitution	*p.Arg273His* (D)
S2 36P	ER− PR− Her2+	c.434T>A	Substitution	*p.Leu145Gln* (D)
S2 37P	ER− PR− NA	c.517G>T	Substitution	*p.Val173Leu* (D)
S2 38P	ER− PR− NA	c.421T>C	Substitution	*p.Cys141Arg* (D)
S2 39P	ER+ PR− Her2+	c.469G>T	Substitution	*p.Val157Phe* (D)
S2 40P	ER+ NA NA	c.707A>C	Substitution	*p.Tyr236Ser* (D)
S2 41P	ER+ NA NA	c.742C>T	Substitution	*p.Arg248Trp* (D)
S2 42P	ER+ PR+ Her2−	c.535C>T	Substitution	*p.His179Tyr* (D)
S2 43P	ER+ PR+ Her2−	c.997C>T	Substitution	*p.Arg333Cys* (D)
S2 44P	ER+ PR+ Her2−	c.401T>C	Substitution	*p.Phe134Ser* (D)
S2 45P	ER+ PR+ Her2−	c.796G>A	Substitution	*p.Gly266Arg* (D)
S2 46P	ER+ PR+ Her2−	c.578A>G	Substitution	*p.His193Arg* (D)
S2 47P	ER+ PR+ Her2−	c.400T>G	Substitution	*p.Phe134Val* (D)
S2 48P	ER+ PR+ Her2−	c.743G>A	Substitution	*p.Arg248Gln* (D)
S2 49P	ER+ PR+ Her2−	c.646G>A	Substitution	*p.Val216Met* (D)
S2 50P	ER+ PR+ Her2−	c.797G>T	Substitution	*p.Gly266Val* (D)
S2 51P	ER+ PR+ Her2−	c.818G>A	Substitution	*p.Arg273His* (D)
S2 52P	ER+ PR+ Her2+	c.402T>G	Substitution	*p.Phe134Leu* (D)
S2 53P	ER+ PR+ Her2+	c.569C>T	Substitution	*p.Pro190Leu* (D)
S2 54P	ER+ PR+ Her2+	c.839G>C	Substitution	*p.Arg280Thr* (D)
S2 55P	NA NA Her2+	c.581T>G	Substitution	*p.Leu194Arg* (D)

Abbreviations: As, malignant ascites; Ax, axilla; Bo, bone; Br, brain; Ch, chest wall; NA, not available; Li, liver; LN, lymph node; Lu, lung; Pl, pleura; Sc, sub-cutaneous; St, soft tissue; Thy, thyroid; ER, oestrogen receptor; PR, progesterone receptor; TNBC, triple negative breast cancer.

Mutations are denoted according to HGVS recommendations (http://www.hgvs.org/). Complex mutations are shown in bold type and simple mutations in plain type. Missense *TP53* mutations are predicted as neutral (N) or deleterious (D) by the Sorting Intolerant from Tolerant (SIFT) and align-GVGD (AGVGD) algorithms as used in the IARC TP53 database (http://www-p53.iarc.fr). The mutation *Pro300Leu* is predicted deleterious by SIFT and neutral by AGVGD and is denoted as N/D.

a Nucleotide numbering reflects cDNA numbering with 1 corresponding to the A of the ATG translation initiation codon in the GenBank reference sequence NM_000546.4.

b The 3 bp deletion affects position 3 (C) of codon 131 and positions 1 and 2 (AA) of codon 132. The reading frame is maintained with amino-acid change from Asn>Lys at 131.

c Homozygous mutation.
